# Supplementary material for: Pathophysiological Significance of Neutrophilic Transfer RNA-Derived Small RNAs in Asymptomatic Moyamoya Disease
Source: Cells. 2021 May 1;10(5):1086. doi: 10.3390/cells10051086 (PMC8147334; doi:10.3390/cells10051086)

2D Structure	Local AU	Position

```
333                    8mer-1a    360
 5'-aagccTTATGAATGC-AGTGAATGTGGGa-3' utr
        |:  |:|  :||  ||    |||||||
 3'-uccuuAGGAUUGGCGAUCUGGUACACCCu-5' tRF
                 16151413   7 6 5 4 3 2
               3' Pairing      Seed
```

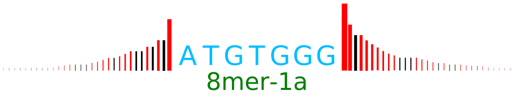

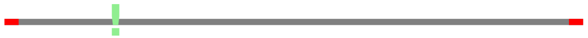

2D Structure	Local AU	Position

```
249                    8mer-1a    276
 5'-agGCATTACAAATGC-AGTGAATGTGGGa-3' utr
      |  ||:   ||  :||  ||    |||||||
 3'-ucCUUAGGAUUGGCGAUCUGGUACACCCu-5' tRF
                 16151413   7 6 5 4 3 2
               3' Pairing      Seed
```

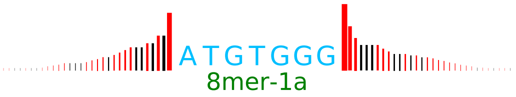

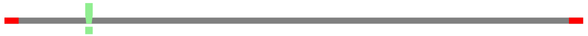

Supplement: Supplementary file 1 [file cells-10-01086-s001.zip › cells-1149161 3rd supplementary materials/Data Supplements/Data Supplement V.zip!/Data supplement 6-mRNAs targets of tsRNAs/tsRNA-Glu-TTC-010/Diagrams/tRF-Glu-TTC-010_vs_NM_001304336(ZNF773).pdf]
